# A Translational Neural Network Mechanism of Resilience: Top-Down Control and Plasticity of the Visual Cortex Relates to Resilient Outcome and Performance

**DOI:** 10.34133/research.1215

**Published:** 2026-04-01

**Authors:** Hendrik Backhaus, Edoardo Pinzuti, Saleh Altahini, Anya Dietrich, Ken-Ichiro Tsutsui, Takuya Sasaki, Keiichi Kitajo, Nicolas Ruffini, Anna Wierczeiko, Margarita Tevosian, Michael Wibral, Albrecht Stroh, Oliver Tüscher

**Affiliations:** ^1^ Leibniz Institute for Resilience Research, Mainz 55122, Germany.; ^2^ MEG Unit, Brain Imaging Center, Goethe University, Hessen 60528, Germany.; ^3^ Institute of Physiology I, University Hospital Münster, Münster, Germany.; ^4^ Laboratory of Systems Neuroscience, Graduate School of Life Sciences, Tohoku University, Sendai, Miyagi 980-8577, Japan.; ^5^ Department of Pharmacology, Graduate School of Pharmaceutical Sciences, Tohoku University, Sendai 980-8578, Japan.; ^6^ Division of Neural Dynamics, National Institute for Physiological Sciences, Okazaki, Aichi 444-8585, Japan.; ^7^ Physiological Sciences Program, Graduate Institute for Advanced Studies, SOKENDAI, Okazaki, Aichi 444-8585, Japan.; ^8^ Institute of Human Genetics, University Medical Center, Mainz 10587, Germany.; ^9^ Department of Psychiatry, Psychotherapy and Psychosomatic Medicine, University Medicine of the Martin-Luther University Halle-Wittenberg, Halle, Germany.; ^10^ Institute of Physiological Chemistry, University Medical Center of the Johannes Gutenberg University, Mainz, Germany.; ^11^ Dynamic Bioimaging Lab, Advanced Optical Microscopy Centre, Biomedical Research Institute, Agoralaan C (BIOMED), Diepenbeek, Belgium.; ^12^ Department Data-driven Analysis of Biological Networks, Georg August University, Göttingen 37077, Germany.; ^13^ German Center for Mental Health (DZPG), Site Halle-Jena-Magdeburg, Halle (Saale), Germany.

## Abstract

To reduce mental disorder prevalence, the understanding of resilience to stress-related disorder and its neurobiological mechanisms has come into the focus of biomedical research to develop both biologically rooted prevention and innovative therapeutic approaches for stress-related disorder. While some resilience mechanisms have been exemplified on the molecular, cellular, and brain-regional level, evidence on the neural systems level is rather sparse. We present the first translational evidence of adaptive plasticity in visual microcircuits and top-down modulation onto the visual system as a neurobiological resilience mechanism at the neural systems level in both humans and mice. In humans, we demonstrate that this adaptive microcircuit plasticity is linked to interactions between neurocognitive domains—executive and perceptual—and between brain regions—frontal and occipital—in specific oscillatory frequencies (β band in frontal inferior frontal gyrus and γ band in occipital V2). Additionally, expanding upon prior resilience research, our findings offer further evidence that phenotypic resilience is associated not only with macro- and microcircuit plasticity but also with better performance in neurocognitive functions central to resilience, i.e., perceptual discrimination in mice and cognitive control in humans. In mice, using awake 2-photon calcium imaging, we observed distinct resilient and susceptible network phenotypes in mouse visual cortex. Resilient animals surpassed both susceptible animals and nonstressed controls in their ability to encode visual afferents. This suggests an improved performance supporting the concepts of posttraumatic growth and stress inoculation on a neurobiological level. Resilience at the neural systems level involves active, dynamic processes rather than being merely passive responses to stress and constitutes a first example that neural network states of resilience are metastable, self-stabilizing, and noncontinuous entities that could serve as a target for new neural network interventions for fostering resilience.

## Introduction

The burden of stress-related mental disorders in western countries is constantly growing, including increasing prevalence in young people [1–4]. Yet, while a substantial fraction of individuals react to a potentially traumatic event (PTE) by the development of a post-traumatic or other stress-related disorder, the majority of traumatic stress-exposed individuals maintain or quickly regain mental stability and functioning, hence showing resilient outcome [5,6]. Investigating those resilient individuals unravels the mechanisms that actively maintain health and function and counteract the transition into disease [7]. Resilience can be conceptualized as a good long-term mental health despite adversity [6]. Operationalized and measured as such an outcome [8], resilience can be investigated translationally in humans and animals, allowing for the identification of not only behavioral but also neurobiological resilience mechanisms. Traditionally, resilience has been defined by its phenotypical representation. Yet, the given behavioral phenotype must be represented by neuronal network dynamics. Here, we asked whether the phenotypic outcome of resilience is reflected by distinct properties of neural network dynamics.

Behavioral paradigms reflecting neurocognitive processes involved in putative neurobiological resilience mechanisms entail, among others, stress reactivity (attentional bias), perceptual discrimination (pattern separation), and cognitive control (aversive system inhibition) paradigms [9]. The emotional Flanker task used here specifically operationalizes the interplay of cognitive stimulus control (Flanker reaction time; aversive system inhibition; here in the visual domain) with (implicit) negative attentional bias and the need to perceptually discriminate visual threatening versus nonthreatening stimuli [10]. We have recently shown that the individual emotional and cognitive interference effects on behavior generated within this paradigm are predicted by information flow both within the right inferior frontal gyrus (rIFG) and from the pars triangularis of the rIFG to parieto-occipital areas (such as the precuneus and V2) in a frequency-specific manner [11]. However, the relation of these neurocognitive processes to an outcome-based measure of resilience has not yet been shown, let alone the potential neural network mechanisms constituting resilience on the neural network level [9].

Hence, here, we used electroencephalography (EEG) recordings from a large cohort of human participants (*N* = 121) and employed source reconstruction with finite-element head modeling during an emotional Flanker task to establish the relation of neurocognitive processes of cognitive-emotional interference control to an outcome-based measure of resilience and assess its neural network bases on a macro-circuit level. To reveal the corresponding underlying neurophysiological mechanisms of resilience on the neuronal micro-circuit level, we chose the well-established chronic social defeat (CSD) mouse model, followed by measuring social interaction (SI) as a measure of resilient outcome. Notably, even in inbred mice, the SI is widely varying between individuals. This indicates that an individualized response of the neuronal network to the stressor resulting in a bimodal classification of animals in stress-resilient versus nonresilient animals is performed [12]. We adopted this classification and employed an SI score threshold of 100 for the differentiation between stress resilient and nonresilient outcomes [13].

Based on the human findings of information flow to (putative to down-modulation of) the parieto-occipital areas including the visual cortex, we hypothesize that stress resilience should be reflected in a cortex-wide functional signature, beyond prefrontal circuits. This should include also primary sensory cortices such as the visual cortex, even more though, as the ability for perceptual discrimination might contribute to a resilient outcome [9]. Therefore, we investigated primary sensory networks in the context of phenotypic resilience, asking whether network resilience constitutes an adaptive network ability. For probing this concept, after subjecting mice to the classical CSD paradigm and the SI test [12], we conducted 2-photon calcium imaging of cortical networks in the awake behaving animal. Assessing both spontaneous and sensory-evoked neuronal activity (perceptual discrimination, i.e., pattern separation) with single-neuron resolution is well suited to derive a fine-grained picture of the local functional architecture [14–16] of a resilient network. By that, we transcend the concept of resilience from the behavioral domain to the neural network domain.

## Results

### Predictive power of emotional interference inhibition on resilience

**Fig. 1.**
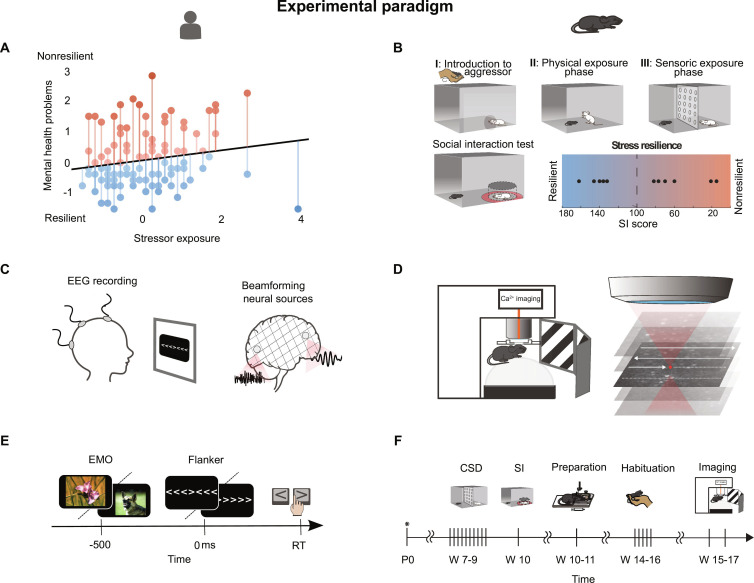
Resilience can be conceptualized in parallel in humans and mice. (A, C, and E) Human study. (B, D, and F) Study in mice. (A and B) Assessment of resilience. (A) Assessment of resilience in human, individual mental health reactivity to stressor exposure [stressor reactivity (SR) proxy score], of 117 subjects. The regression line shows the normative linear positive relationship between exposure to Life events (LEs) stressors and mental health problems. The residuals onto the regression line are subjects’ deviations from the normative stressor exposure–mental health problems relationship. A strong positive deviation reflects high susceptibility of the subject’s mental health to the effects of LEs (high SR, high SR proxy score); a strong negative deviation reflects below-average low susceptibility (low SR proxy score). (B) Assessment of resilience in mice via a testing social interactions (SIs) after exposure to an aggressor in a chronic social defeat (CSD) paradigm. (C and D) Neurophysiology. (C) EEG recording and beamformer source reconstruction in the human EEG cohort. (D) Ca^2+^ imaging in mice during visual stimulation via drifting gratings of different orientation. (E and F) Experimental design. (E) Performance of the Emo–Flanker task. (F) Schedule in weeks (W) for resilience testing in mice: CSD paradigm (CSD), SI test (SI), Preparation for Ca^2+^ imaging, Habituation to imaging setup, Ca^2+^ imaging.

**Fig. 2.**
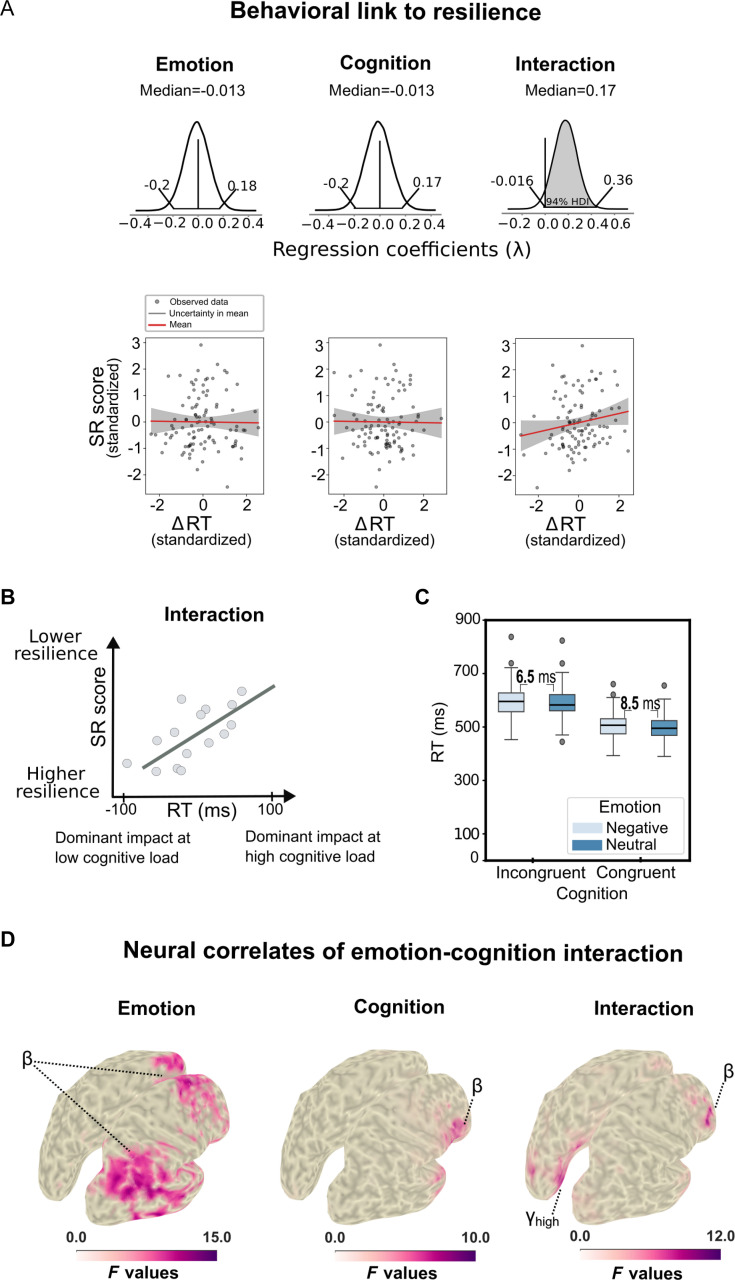
SR score relates to behavioral measures and source activity. (A) Posterior distributions of the regression coefficients (λ) and their 94% highest density intervals (HDIs) and the corresponding regression plots for the Bayesian linear regression of SR proxy scores against reaction time (RT) differences for neutral versus negative emotional stimuli (ΔRT_emotion_) (left), congruent versus incongruent Flanker stimuli (ΔRT_cognition_) (middle), and the interaction effect in reaction times [ΔRT_interaction_ = (ΔRT_Incong_. _Neg._ − ΔRT_Incong. Neut._) − (ΔRT_Cong. Neg._−ΔRT_Cong. Neut._)] (right). Colored regions mark the part of the distribution below or above zero that contains the 94% HDI. If the HDI contains zero, the largest effect is highlighted in gray. (B) Conceptual explanation of the negative interaction effect as parameterized by the contrast in our study. (C) Detailed post hoc analysis of the interaction effect—The reaction time increase induced by negative emotional stimuli is higher in the congruent than in the incongruent condition. Boxplots show subject-level reaction time distributions (median, interquartile range (IQR), 1.5× IQR whiskers; dots indicate outliers). (D) Statistical maps of main and interaction effects at the level of source reconstructed neural activity. β, β-band activity (9 to 33 Hz); γ_high_, high-frequency (64 to 140 Hz) γ-band activity (adapted from [11]).

We investigated whether baseline behavioral measures of emotional interference inhibition derived from the Emo–Flanker task, as an operationalization of the putative neurocognitive resilience mechanisms stress reactivity (attentional bias), perceptual discrimination (pattern separation), and cognitive control (aversive system inhibition), could predict the stressor reactivity (SR) proxy score as our resilience measure (see Fig. 1A and Human EEG—operationalization and measurement of resilience in human subjects section). Therefore, we assessed 2 distinct Bayesian regression models: one incorporating reaction times (representing the stimulus interference inhibition component, i.e., visual cognitive stimulus control) and the other incorporating accuracy metrics (representing the response inhibition component, i.e., executive inhibitory control) as predictors for the SR proxy score [12]. For reaction times, the Emotion predictor (Negative − Neutral conditions) revealed no correlation with the SR proxy score proxy λ = −0.013, [−0.2, 0.18]. No significant effect was also observed for the Cognition predictor (Incongruent − Congruent) λ = 0.013, [−0.2, 0.017]. In contrast, the Interaction predictor [(Incongruent Negative − Incongruent Neutral) − (Congruent Negative − Congruent Neutral)] exhibited a significant positive correlation with the SR proxy score proxy λ = −0.17, [−0.016, 0.36]. For accuracy, the Emotion and Cognition predictors had no relationship with the SR proxy score λ = 0.00, [−0.19, 0.2] and λ = 0.00, [−0.26, 0.26], respectively. The Interaction predictor had only a partial negative relationship with the SR proxy score, λ = −0.12, [−0.38, 0.14] (see Fig. S1). By analyzing the relationships between these behavioral measures and the SR proxy score, we identified visual cognitive stimulus control (emotional interference inhibition component as measured by reaction times predictor) as critical in forecasting resilience. Specifically, for the Interaction effect, subjects exhibiting higher reaction time disparities in the high cognitive load condition tend to have higher SR proxy scores, i.e., lower resilience (see Fig. 2B and C).

### The influence of emotions on stimulus processing primarily affects perceptual and executive areas in the human brain

Having established at the behavioral level that the stimulus interference inhibition component of the Emo–Flanker task predicts the SR proxy score, we now aimed to investigate whether the brain network activity involved in this task can also predict resilience scores. Therefore, we recap our previous results [14–16] and then focus on the neurophysiological findings that correlate with resilience.

We have previously determined significant neural sources in the Emo–Flanker task utilizing a 2 × 2 cluster permutation analysis of variance (ANOVA) with 3 factors: Emotion (Negative versus Neutral), Cognition (Incongruent versus Congruent), and the Interaction of Emotion and Cognition [(Incongruent Negative − Incongruent Neutral) − (Congruent Negative − Congruent Neutral)] [14–16]. The results can be delineated into 3 pivotal observations. Firstly, a widespread activation was observed in the brain during the main effect of emotion in the β band (see Fig. 2D, left) and the γ band. This primarily affects the frontal regions, with considerable influence on the parietal and occipital lobes. In contrast, only a few regions, such as the IFG, demonstrated significance in the main effect of cognition. Secondly, the rIFG was the sole source exhibiting significant activity modulation across all 3 contrasts (*n* = 103 participant datasets in final analyses): the main effects of emotion (rIFG pars orbitalis, Montreal Neurological Institute (MNI) peak coordinates *x* = 45, *y* = 40, *z* = 0, *F* = 10.4, *P* < 1.9996e−04), cognition (rIFG pars opercularis, MNI peak coordinates *x* = 55, *y* = 10, *z* = 10, *F* = 7.9, *P* < 1.9996e−04), and the interaction effect (rIFG pars triangularis, MNI peak coordinates *x* = 45, *y* = 40, *z* = 20, *F* = 9.6, *P* < 1.9996e−04)—all occurring in the β band (Fig. 2D). The activation of the rIFG (in its 3 subdivisions) in all 3 contrasts underscores its pivotal role in modulating emotion, cognition, and their interaction effects. Thirdly, in the interaction contrast, 2 posterior sources, namely, the precuneus (*n* = 103, MNI peak coordinates *x* = −5, *y* = −60, *z* = 30, *F* = 7.8551, *P* < 1.9996e−04) and visual area V2 (*n* = 103, MNI peak coordinates *x* = 5, *y* = −80, *z* = 20, *F* = 7.9948, *P* < 1.9996e−04), exhibited a significant emotion–cognition interaction effect (refer to Fig. 2D, right high γ band).

Together, these results suggest that the Emo–Flanker task, as an operationalization of the putative neurocognitive resilience mechanisms including stress reactivity (attentional bias), perceptual discrimination (pattern separation), and cognitive control (aversive system inhibition), activates a widespread network, extending from frontal areas to parietal and occipital regions. Interestingly, we identified the IFG as a critical hub in the interaction between emotion and cognition, along with 2 occipital areas. These identified neural sources will serve as the foundation for linking SR proxy scores and network activity in the subsequent analysis.

### Top-down modulation from the rIFG to the visual cortex and power spectra interference in the human brain predict resilience

**Fig. 3.**
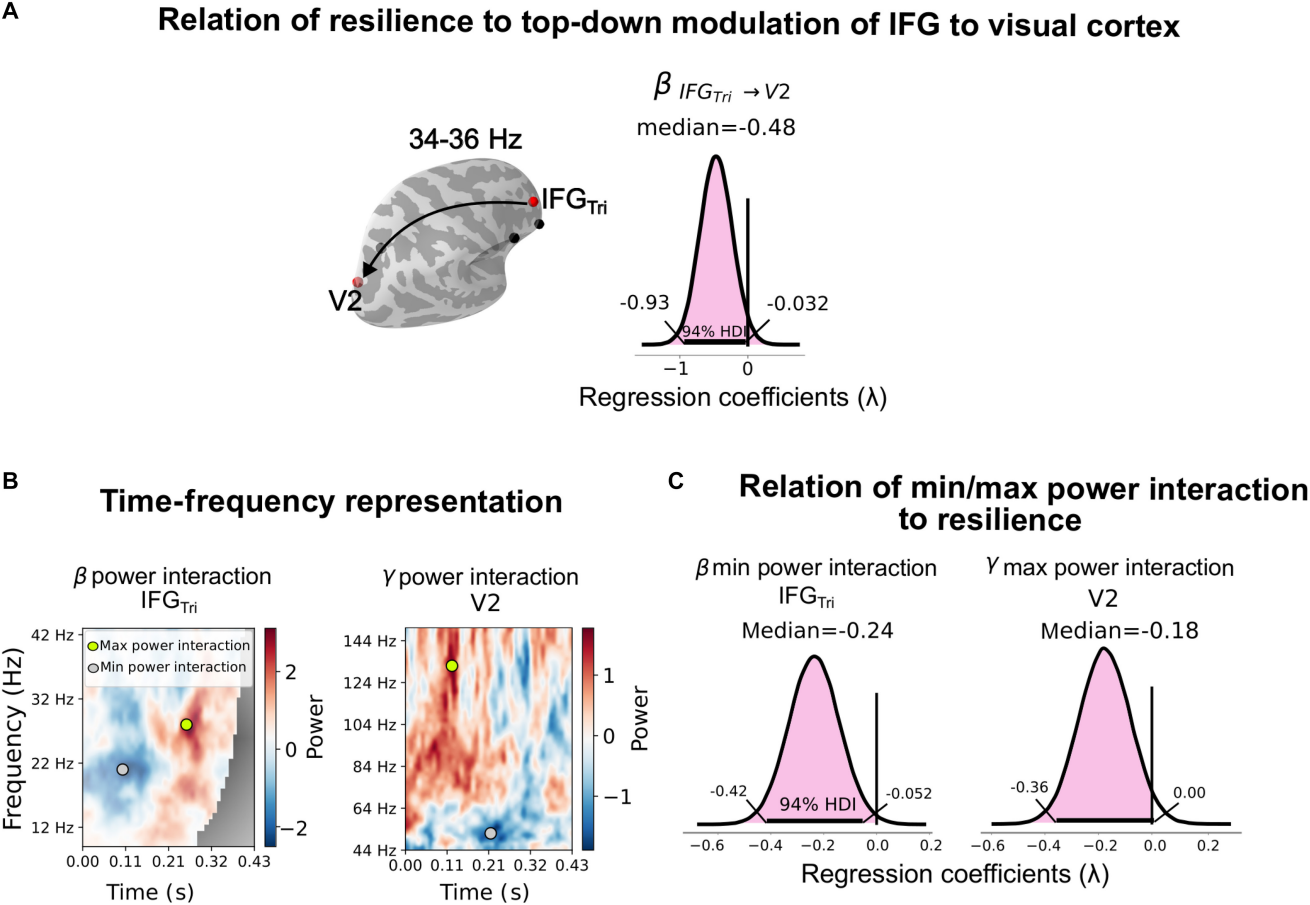
SR proxy scores impact long-range information flow and statistical interaction effects in spectral power at the source level. (A) Posterior distributions and 94% HDI for Bayesian linear regression of SR proxy scores against various long-range information flows in multiple frequency bands (α, β, γ) assessed by Granger causality (GC) from IFG_Tri_ to V2; only the most influential regression coefficient is shown. (B) Interactions [i.e., double differences, (Incongruent Negative − Incongruent Neutral) − (Congruent Negative − Congruent Neutral)] in β- and γ-band spectral power in an individual subject. Colored dots mark the time–frequency locations of the numerically lowest and highest interactions. The corresponding values for each subject entered the regression analysis in (C). (C) Posterior distributions and 94% HDI for Bayesian linear regression of the SR proxy score against interactions in the spectral power in the time–frequency domain.

Next, we aimed to investigate whether a top-down interaction from the frontal rIFG subdivisions to the occipital areas predicts the SR proxy score. Thus, we employed a Bayesian linear regression with the 7 significant Granger causality (GC) links, found in [14–16], as predictors. In Fig. 3A, we reported the result with the highest effect (all predictors results can be found in Fig. S2).

The long-range connectivity (GC) IFG_Tri_ to visual cortex area 2 (V2) had the strongest negative relationship with the SR proxy score λ = −0.48, [−0.93, −0.032], while the GC link IFG_Tri_ to precuneus had a small positive effect λ = 0.26, [−0.14, 0.67] (see Fig. S2). None of the other GC links showed any relationship with the SR proxy score (see Fig. S2). To determine whether IFG_Tri_ and V2 are key regions for predicting SR proxy score, we performed a Bayesian linear regression analysis of identified minimum and maximum β-power interaction (in the range of 10 to 44 Hz) for IFG_Tri_ and γ-power interaction (in a frequency range of 44 to 160 Hz for V2) (see Fig. 3B). Multiple Bayesian models were evaluated, including models with only minimum and maximum β power for IFG_Tri_, minimum and maximum γ power for V2, a model with only minimum IFG_Tri_ β-band and maximum V2 γ-band activity, and a full model with all predictors (see Methods). After comparing these models, we found that the combined model—using only the minimum β-power interaction in the rIFG_Tri_ and the maximum γ power interaction in V2—provided the strongest predictive accuracy for SR proxy score. In this final model, both predictors demonstrated a strong negative effect on SR proxy score, with the minimum β-power interaction in IFG_Tri_ [λ = −0.24, confidence interval (CI) = [−0.42, −0.052]] and the maximum γ-power interaction in V2 (λ = −0.18, CI = [−0.36, 0.0079]) showing significant associations (see Fig. 3C). These results suggest that the activity in these specific frequency bands in IFG_Tri_ and V2 is critical for accurately predicting SR proxy score (for full model comparisons results, see Table S1).

In this analysis, we tested whether there is a link between the SR proxy score and network activity in terms of top-down modulation. We found that lower long-range connectivity (GC) activity from IFG_Tri_ to V2 is associated to a higher SR proxy score, i.e., lower resilience, indicating that the strength of the top-down modulation in the β band to occipital is predictive of resilience. To corroborate these findings, we examined individual sources (IFG_Tri_ and V2) by analyzing their minimum β power and maximum γ power. We found that oscillatory activity within these frequency bands also correlated with SR proxy score. The negative relationship between the minimum β-power interaction and SR proxy score indicates that a higher inhibitory activity in the IFG_Tri_ is associated with a higher individual resilience (see Discussion).

Hence, we established the association of resilience with fronto-occipital β-oscillatory modulation, indicating a pivotal role for visual perceptual processing. The behavioral association of a reaction time interference effect with resilience and this macro-circuit modulation hinted at an involvement of the neurocognitive resilience mechanism of perceptual discrimination. Therefore, we ask the question if there is a micro-circuit correspondence within the visual system. For that, we used the well-established CSD mouse model paradigm in combination with the micro-circuit neuroimaging and a visual discrimination task as described in the following paragraphs.

### Combining CSD and SI with 2-photon neuronal microcircuit recordings in the visual cortex in awake mice

We subjected male BL6 mice to a CSD paradigm to mimic severe stress exposure. The experimental animals were exposed to a daily changing aggressor over 10 consecutive days (Fig. 1B, I to III). The experimental mouse remained in the aggressors’ cage for the following 24 h. Subsequent to the CSD, an SI test was carried out (Fig. 1B). As a proxy for stress resilience, the SI score was calculated (see Methods: Animal—CSD and SI test). A high SI score signifies that the mouse spent comparably more time in proximity to the separated aggressor. This behavioral signature has been associated with higher stress resilience, with the mouse being able to differentiate between the contexts [12,17]. The mouse perceives that the aggressor is not posing a thread and engages in an SI. The SI scores of the 11 animals included in the study ranged between 15 and 162 (Fig. 1B). The SI scores were also assessed from the control group, which did not undergo prior CSD (Table S1). It is important to note that the surgical procedures required for longitudinal 2-photon calcium imaging were performed after the CSD/SI. This is to avoid any impact on social behavior and interaction by either the surgery or the head holder (Fig. 1D). We focused on the recordings of local cortical microcircuit activity in the primary visual cortex (V1), specifically expressing the genetically encoded calcium indicator GCaMP6f in excitatory neurons. The animals were allowed to recover for 4 weeks post-surgery to ensure stable and strong GCaMP6f expression [18]. Importantly, the animals were carefully habituated to the head fixation. They did not display any signs of discomfort throughout the imaging experiments (Fig. 1F). We proceeded to conduct functional fast full-field 2-photon calcium imaging in the awake animal, utilizing a spherical polystyrene treadmill to assess locomotion during the experiments (Fig. 1F). The animal was surrounded by a 270° monitor ring, for providing visual stimulation. Intracellular calcium fluctuations were detected and served as a proxy for the identification of putatively action potential (AP)-related calcium transients in layer II/III of V1 [19,20] (Fig. 1F).

### Resilient mice exhibit lower spontaneous activity levels in neuronal microcircuits of the primary visual cortex

**Fig. 4.**
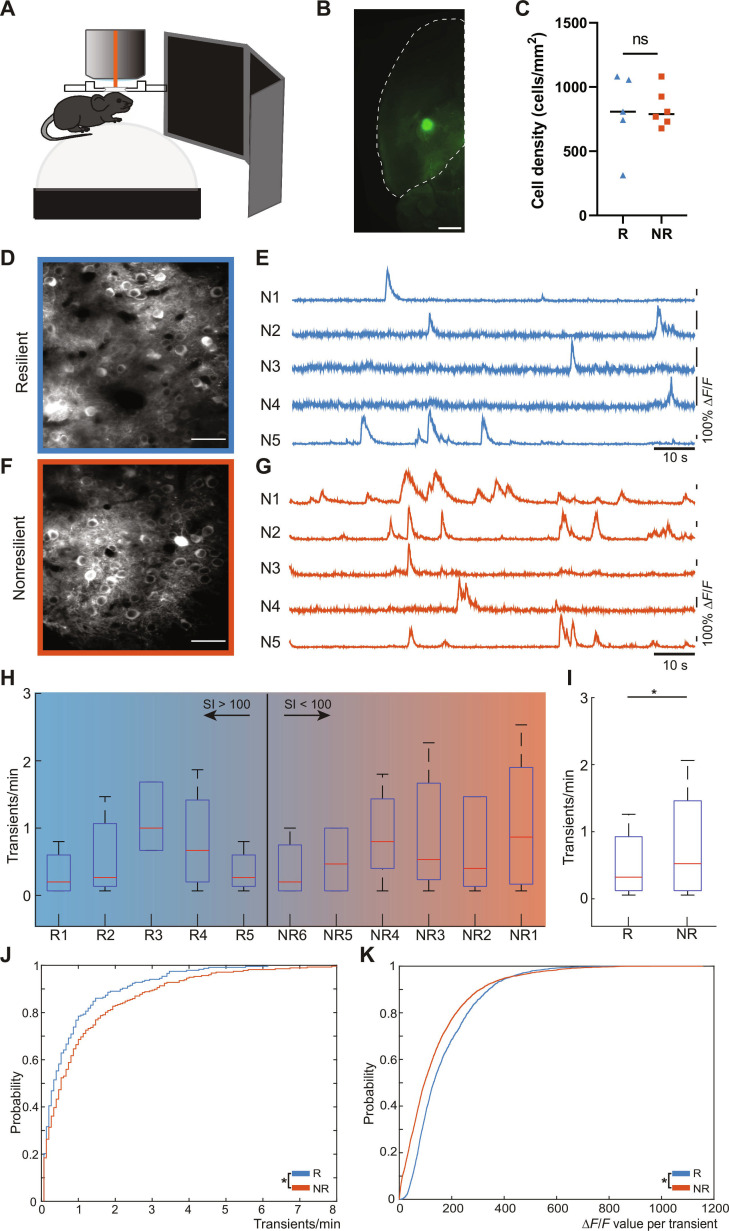
Local network activity in V1 reflects resilience. (A) Schematic illustration of assessment of spontaneous ongoing neuronal activity. Surrounding screens were turned off during initial measurement to guarantee the absence of visual stimuli. (B) Light sheet microscopy image showing the injection site of GCaMP6f expression. The injection was performed −3 mm posterior and −2.5 lateral from bregma. (C) A comparison of recorded cell densities reveals no significant difference between micrographs recorded in resilient versus nonresilient (2-sided *t* test, R versus NR = 0.8283). (D and F) Micrographs of microcircuits in layer II/III of the primary visual cortex, recorded by 2-photon microscopy, of a resilient (D) and nonresilient (F) animal (scale bars, 50 μm), and the corresponding intensity traces of somata within the microcircuit (E and G). (H) Boxplot of the activity frequency per animal in relation to their SI value. Whiskers indicate 5th to 95th percentiles. (I) Boxplot of the activity frequency of all recorded somata pooled together in resilient and nonresilient groups, considering *n* = 237 somata (R) and *n* = 277 somata (NR), respectively. Whiskers indicate 5th to 95th percentiles. (J) A significant difference in the cumulative distribution of activity frequencies is observed (2-sided Kolmogorov–Smirnov test, *P* = 0.0478). (K) Cumulative distribution of Δ*F*/*F* amplitude of individual calcium transients. Resilient mice exhibit significantly lower amplitudes per transient compared to nonresilient mice (*P* = 2.5328e−52, 2-sided Kolmogorov–Smirnov test).

In all animals included in this study, we could identify the functional signature of local networks in layers II/III of V1 (Fig. 4D to G). We did not observe any differences in terms of cellular density between the animals (Fig. 4C). No signs of elevated cellular disintegration could be observed either. The excitatory neurons exhibited typical sparse spontaneous calcium transients, passing our criteria for putative AP-related events [18]. We quantified the average activity rate on a single neuron basis. Based on the beforementioned classification, we grouped the animals in the resilient and nonresilient categories (Fig. 4H) and found a significant positive shift in cortical microcircuit activity (Fig. 4I and J). The amplitude of calcium transients showed a similar shift between resilient and nonresilient animals (Fig. 4K). Measurements of the same microcircuit were performed 1 week later in a resilient and a nonresilient mouse. We compared the activity levels of all neurons within an animal that could be colocalized in both measurement time points. Single neurons comprised a shift in the activity rates, but the activity signature of the microcircuit in its entirety did not change, independent of the behavioral outcome of chronic stress exposure (Fig. S3). This suggests that the functional architecture remains at least metastable.

### Nonstressed animals exhibit activity dynamics close to the dynamics of nonresilient animals

**Fig. 5.**
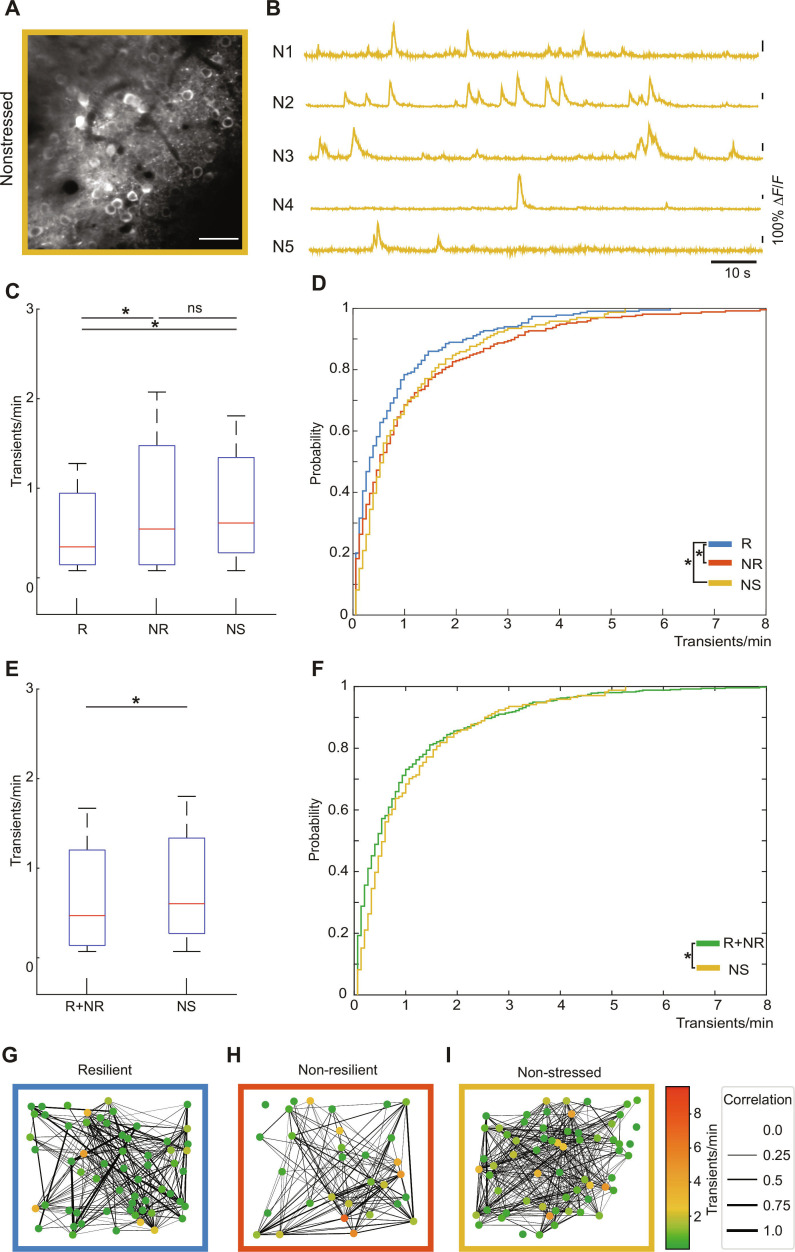
Nonstressed animals show similar activity levels as nonresilient animals. (A) Recorded micrograph of a nonstressed animal (scale bar, 50 μm), and (B) the extracted intensity traces of areas in the field of view containing neuronal somata do not show any morphological difference to the 2 stressed groups. The average cell density was 66 ± 7 neurons. (C) Boxplots and (D) cumulative distributions of activity frequencies of all somata pooled in a nonstressed group considering that *n* = 171 somata show no difference toward the group of nonresilient somata but significantly differ to the resilient group (2-sided Kolmogorov–-Smirnov test, *P* = 0.0004, *n* = 171 somata). Whiskers indicate 5th to 95th percentiles. (E) Boxplots and (F) cumulative distributions of activity frequencies of all somata of the resilient and nonresilient group show a significant difference to the nonstressed group (2-sided Kolmogorov–Smirnov test, *P* = 0.0067). Whiskers indicate 5th to 95th percentiles. (G to I) Connectivity maps of the neuronal ensembles during task free, spontaneous activity of a resilient (G), nonresilient (H), and nonstressed (I) animal. A significant difference in distributions of correlation coefficients within neuronal networks of all animals, pooled into the stratified groups of resilient, nonresilient, and nonstressed, is observed (R versus NR: *P* = 5.36e−28, R versus NS: *P* = 4.69e−45, and NR versus NS: *P* = 1.28e−54, 2-sided Kolmogorov–Smirnov test).

We now asked whether the network dynamics of nonstressed animals, i.e., animals that never went through the CSD paradigm, is similar to resilient or to nonresilient animals, or whether it represents another—third—distinct network state. For that, we subjected mice to the SI test, which did not undergo the CSD paradigm. In all other aspects of the experimental procedures, nonstressed mice underwent the same procedures as their stressed mates. The recorded microcircuits (Fig. 5A) exhibited a neuronal density very similar to the densities of the resilient and nonresilient groups. As in both other conditions, typical sparse activity of putatively AP-related calcium transients was observed (Fig. 5B). The activity frequencies of neurons within microcircuits of nonstressed mice were compared to the results of treated groups. We found that the nonstressed group rather resembles the nonresilient group. Indeed, the resilient group displayed activity dynamics significantly differing from both the nonstressed and the nonresilient group (Fig. 5C and D). To assess if these new properties of the network emerge upon stress exposure or is it simply a stratification that had been present in the pre-stressed population, we pooled the activity signatures of both the resilient and nonresilient group (in the absence of pre-stressed measurements, which had been omitted to not impact the CSD/SI paradigm by the presence of the head holder and the cranial window). When comparing these 2 stressed groups to the nonstressed controls, a significant difference still remains both on the level of the mean activity state and on the level of the activity distribution (Fig. 5E and F).

Conducting imaging-based functional recordings such as 2-photon calcium imaging affords the assessments of network topologies [16]. We analyzed functional connectivity between neurons while at the same time encoding for their respective activity state (Fig. 5G to I). In the resilient group, the network is characterized by overall high connectivity, with a few highly interconnected hubs. This architecture is generally associated with an efficient network topology in terms of information transfer and processing [21]. In contrast, nonresilient networks are rather characterized by a uniform connectivity, with decreased connection strengths, and a significantly different connectivity matrix. This is surprising at first sight, as the overall activity state of the susceptible networks is increased [22]. Yet, particularly the highly active neurons seem to be rather poorly connected. Consequently, the high activity of these neurons seems to be rather detrimental to overall efficient network function, suggesting a maladaptive state. The nonstressed controls follow the topological organization of resilient networks.

### Resilient microcircuits surpass nonresilient and nonstressed microcircuits in the accuracy of the representation of visual afferents

**Fig. 6.**
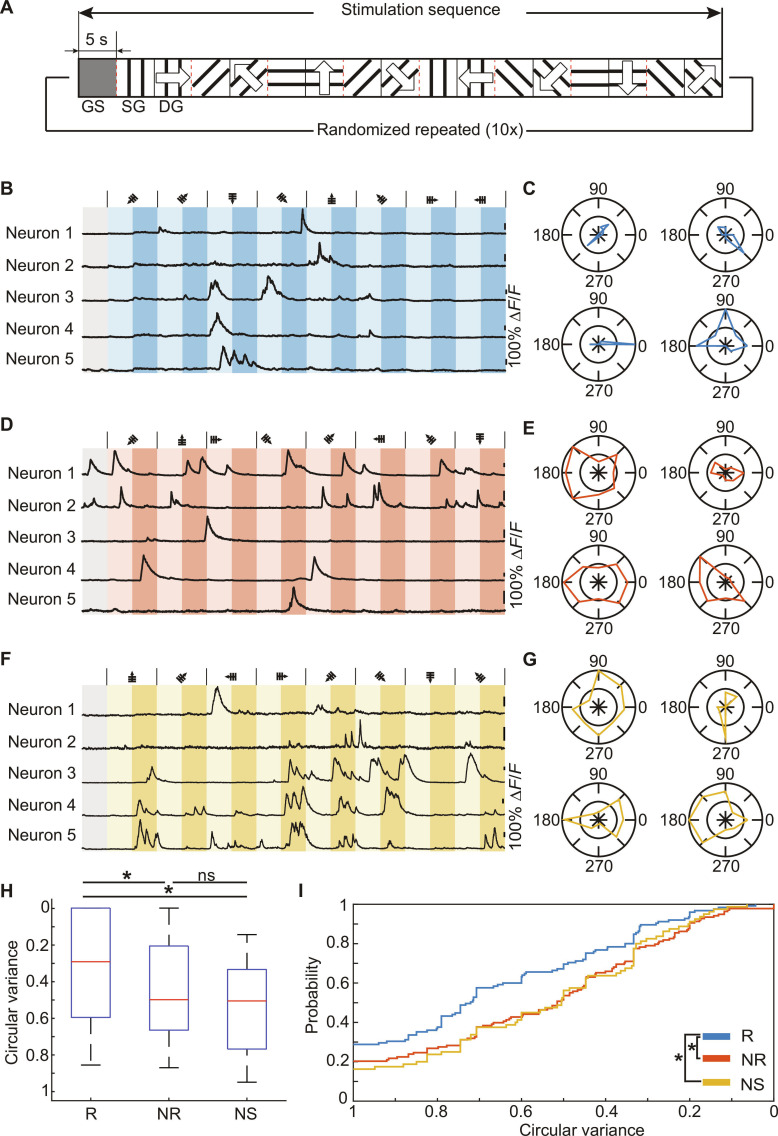
Resilient animals outperform nonresilient and nonstressed mice in processing visual afferents. (A) A paradigm of static and drifting grating presentations is used for visual stimulation. The stimulation sequence is initialized by a 5-s gray screen presentation, followed by a randomized sequence of 8 grating directions (0°, 45°, 90°, 135°, 180°, 225°, 270°, and 315°), first displaying a static and then a drifting grating representation, each lasting 5 s. The sequence is presented 10 times, each time with a new randomization. (B, D, and F) Representative intensity traces recorded during visual stimulation of one sequence for a resilient (B), nonresilient (D), and nonstressed (F) animal and their corresponding polar plots of neuron response functions (C, E, and G). (H and I) The circular variance of each neuron is calculated, and all neurons are pooled into the corresponding 3 groups. Resilient neurons comprise a significantly higher proportion of well-tuned responses compared to nonresilient (*P* = 0.0064) and nonstressed neurons (*P* = 0.0001). No significant difference between nonresilient and nonstressed neurons can be detected (2-sided Kolmogorov–Smirnov test). Whiskers indicate 5th to 95th percentiles.

We then asked whether the unique network activity signature in resilient mice is adaptive or maladaptive with regard to the representation of visual afferents. We employed a static and drifting grating stimulation paradigm while simultaneously assessing single-cell activity in layer II/III of V1 (Fig. 6A). Based on the results shown above, the question arises if a modulatory effect upon chronic stress exposure is leading to an alteration of activity patterns in resilient mice. However, to address the issue, if the effect is adaptive or maladaptive, stimulus free network behavior does not lead to an answer. We found a significant increase in the proportion of neurons in microcircuits of resilient mice displaying a fine-tuned response upon drifting grating stimulations compared to the microcircuits of nonresilient and nonstressed mice. Concurrently, microcircuits of nonresilient and nonstressed mice comprise a similar number of identified neurons exhibiting a broadening of orientation selectivity tuning (Fig. 6H and I).

### Lower spontaneous visual network activity in mice and humans

**Fig. 7.**
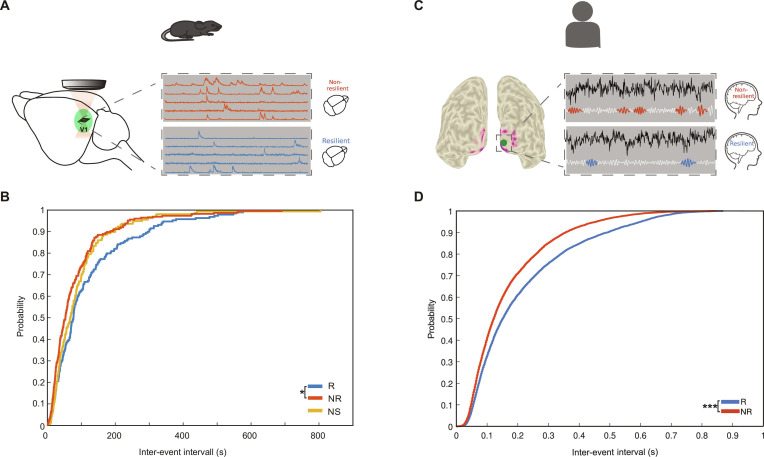
Both in man and mouse, resilience is associated with very similar neuronal network states, characterized by an altered inter-event interval (IEI). (A) Representation of the brain area of data collection in the mouse model (left). The neurons within the field of view (right) are analyzed in terms of IEIs. (B) Cumulative distribution of IEI in resilient (blue), nonresilient (red), and nonstressed (yellow) groups. (C) Two examples of visual source time series (black signal) with identification of burst activity on filtered γ signal (white) for a resilient subject (blue) and nonresilient subject (in red). (D) Cumulative distribution of IEI in resilient (blue) and nonresilient (red) groups.

We further investigated local micro-circuits networks within V1, where the individual neurons were characterized with respect to their activity intervals (Fig. 7A). Here, the inter-event intervals (IEIs) were calculated for each neuron as a measure of the relation of silence periods with periods of neuronal activity.

We found longer IEIs in resilient compared to nonresilient and nonstressed animals (2-sided Kolmogorov–Smirnov test, *P* < 0.001), indicating a lower burstiness of neuronal activity within the group of resilient mice (Fig. 7B). Using graph theory analyses, resilient mice showed a more pronounced and denser global micro-circuit activity compared to nonresilient mice (Fig. 7B, insets). In sum, both measures indicate an adaptive, plastic reorganization of neural microcircuits in resilient mice.

We then went back to our human data and examined whether human resilient and nonresilient subjects also exhibited signs of different cortical excitability in the visual cortex during baseline EEG recordings. To this end, we identified γ-burst events in the parcellated visual areas V1 and V2 and quantified cortical excitability by computing the IEI—the time elapsed between successive bursts. Consistent with findings from the animal model, resilient individuals showed significantly longer IEIs compared to nonresilient subjects (2-sided Kolmogorov–Smirnov test, *P* < 0.001; see Fig. 7D), suggesting lower spontaneous γ activity and excitability in the visual cortex.

## Discussion and Conclusion

### Neurobiological resilience and performance gain through visual microcircuits plasticity and fronto-occipital β- on γ-oscillatory modulation

In the context of the failure to reduce mental disorder prevalence by pathophysiological oriented research [5] and “facing a second pandemic of mood and anxiety disorders” [23] induced by major disruptive events in recent years (COVID pandemic, wars, climate change), the understanding of resilience to stress-related disorder [24] and its neurobiological mechanisms [5,23] has come into the focus of biomedical research to develop both biologically rooted prevention and innovative therapeutic approaches for stress-related disorder [5,23,25].

Neurobiological research on all scalar levels of biology is crucial for unraveling the mechanisms of resilience [5,9,23,26]. On the neuroanatomical level, studies in both humans and laboratory animals have identified several key brain regions and systems involved. Prominently featured are the hippocampus**,** the prefrontal cortex (PFC), and the reward system [9]. Less attention has been paid to other brain systems such as the perceptual/sensory system as well as to the cross-talk between the putative neurocognitive domains and brain systems of resilience, especially the information flow between perceptual (occipital, parietal, and temporal areas) and executive (frontal) brain systems. Resilience is also increasingly understood as an active process mediated by distinct molecular, cellular, and circuit adaptations. These adaptations include changes in neuroplasticity, the brain’s ability to reorganize, as well as molecular, e.g., transcriptional and epigenetic, mechanisms [26]. While some active adaptive changes have been exemplified on the molecular {e.g., KCNQ [potassium voltage-gated channel subfamily Q (genes encoding Kv7 channels)] channels in the ventral tegmental area (VTA)} and cellular level (e.g. neurogenesis in the hippocampus), evidence on the neural microcircuit level and in relation to communication between brain systems (macrocircuit level) is rather sparse (e.g. [27,28]).

Here, we provide first translational evidence for adaptive plasticity of visual microcircuits and top-down microcircuit modulation of the visual system as a neurobiological resilience mechanism on the neural systems level in men and mice. We show on the human level that microcircuit adaptive plasticity relates to the cross-talk between neurocognitive domains (executive and perceptual) and brain systems (frontal and occipital) in certain oscillatory domains (frontal IFG_Tri_ in β band and occipital V2 in γ band). Furthermore, going beyond previous resilience studies, our data also provide additional evidence that there is, beside the relationship of phenotypic resilience with neural macro- and microcircuit plasticity and related to both, a better behavioral performance in neurocognitive domains of putative importance in resilience processes, namely, perceptual discrimination (pattern separation) and cognitive control (aversive system inhibition) in resilient mice and men, respectively. This indicates that resilience processes on the neural system level are active processes and not mere passive adjustments to stress as previously shown on the molecular level [26], and they can lead to a behavioral gain of function—at least in the mouse model—a phenomenon described as posttraumatic growth [29] or stress inoculation [30].

### Microcircuit plasticity in the visual cortex may potentially be induced by a short-term neuronal mechanism in resilient mice

Both instances of resilience-related plasticity of visual microcircuits, i.e., lower spontaneous activity (Fig. 4), including longer IEIs (Fig. 7B) with a more pronounced and denser global micro-circuit activity (Fig. 7B, inset) and more well-tuned task-related neuronal responses (Fig. 6), are present at least 5 to 6 weeks after the induction of resilience by the CSD paradigm in our study; hence, they are meta-stable in an at least intermediate time frame. However, due to the temporal design of our study, we cannot draw any conclusions to the molecular and cellular mechanisms inducing microcircuit plasticity during the CSD paradigm. Intriguingly, Li et al. [27] showed for the auditory system, using the very same CSD paradigm, that in the primary auditory cortex (temporal area A1), parvalbumin (PV) interneurons are activated by a short-term hyperpolarization of thalamic inputs that elicits brain-derived neurotrophic factor–tropomyosin receptor kinase B (BDNF–TrkB)-dependent presynaptic synaptogenesis to promote resilience. The authors observed this effect at the very beginning of the CSD paradigm (day 1), while they found lower A1 activity after 10 d as measures by c-Fos intensity in layers 2/3 to 4 in resilient mice versus controls—resembling our meta-stable findings of lower activity in the visual cortex. However, this interplay between a macrocircuit (cortico-thalamic) and microcircuit (L2/3 presynaptic BDNF–TrkB signaling) mechanism [27] may not be the only (micro-)circuit plasticity mechanism. Social stress resilience also includes aspects implicated in the function of sensory cortical areas, which are critically important in several aspects of resilience. One aspect is sensory discrimination. If an animal—or a human being—is better in identifying a sensory context, a point in the sensory parameter space, it—or he or she—may be better equipped for discerning a context that is directly associated to a previous social stressor, in contrast to a nonthreatening environment. These sensory parameter spaces also include the visual environment. Visual afferents, along the visual path, are cortically first represented in the primary visual cortex. Within the primary visual cortex, particularly layers II/III are highly interconnected and tasked with the representation, computation, and dissemination of the visual parameter space. How accurate the visual parameter space is encoded can be measured by assigning the accuracy of tuning for a given movement. Orientation tuning is a key parameter, very sensitive toward, e.g., early impacts of neurodegenerative disorders [31–33]. Here, we did not focus on early signs of disease. We asked whether a social resilient animal might be better equipped to map the visual parameter space. Indeed, we found that resilient animals generate a more precise representation of the outside visual world, which, in turn, informs higher-order circuits, and which can then differentially affect the phenotypic response. Indeed, particularly in the field of stress resilience, one of the resilience mechanisms might be a better discrimination ability [34–38]. Very recent studies in post-traumatic stress disorder (PTSD) in humans indeed suggest that visual cortical structural covariances are negatively associated with PTSD symptoms, pointing toward the important role of sensory cortical structure and function in resilience and beyond [39]. To discern the mechanisms of local mechanism of resilience-related microcircuit plasticity in the visual domain, future studies with a complementary temporal design (e.g., [27]) are needed.

### Macrocircuit modulation of γ-band visual cortex activity and spontaneous γ-band burst activity are predictive of resilience in humans

On the macrocircuit level, we found that the strength of the top-down modulation by frontal β band onto occipital γ-band oscillatory activity is predictive of resilience in a task-dependent manner (Figs. 2 and 3). In addition, baseline γ-band burst activity was lower in more resilient individuals (Fig. 7D)—comparable to a lower spontaneous single-cell activity in resilient mice. This indicates that the state of visual cortex γ-band oscillatory activity in humans is, in addition to the single-cell activity shown in mice, a determinant of a resilient state on the neural network level. But how, potentially, do the observed neural activity determinants of the macro-circuit-level (γ-band oscillatory activity) and micro-circuit-level [lower (excitatory) single-cell activity] intersect mechanistically? An intriguing possibility may again involve the PV interneurons. PV interneurons are one class of interneurons that are crucial for controlling γ-band oscillatory activity in a broad range of neural networks [40], also in the visual cortex in connection to perceptual discrimination behavior [41]. Through fast perisomatic inhibition, PV interneurons exert powerful inhibitory gating that structures population-level synchrony while suppressing unstructured excitatory firing [42]. Intriguingly, PV + neurons sense (and are able to influence) various frequency bands generated by cortical networks: from δ to γ [43]. Thus, this inhibitory gating mechanism is able to induce both the reduced spontaneous neuronal (somatic) activity observed in mouse calcium recordings (slow wave bursts) and the lower γ burstiness observed in human EEG. Importantly, such PV-mediated inhibitory control not only stabilizes network dynamics but also can enhance circuit performance by increasing the signal-to-noise ratio and promoting more efficient, task-relevant neural processing. Together, these findings suggest that enhanced PV-mediated inhibition may represent a conserved circuit-level signature of resilience across species [44,45]. In this context, the correspondence between rIFG dynamics in humans and prefrontal activity in rodents should be understood as functional rather than anatomical, reflecting shared principles of top-down inhibitory control rather than direct structural homology. Consistent with this interpretation, PV interneurons have recently been implicated as key excitatory/inhibitory-balance regulators in the CSD mouse model paradigm for resilience in intermediate range (thalamo–A1 cortical) [27] and long-range (amygdala–dorsomedial) PFC [28], determining resilient behavior. Human multi-modal neurophysiological assessments [e.g., EEG/magnetoencephalography (MEG) with diffusion tensor imaging (DTI)/structural magnetic resonance imaging (sMRI)] and modelling informed by translational neural mass models [46] will help to test this hypothesis on the macrocircuit and whole-brain level.

### Macro- and microcircuit oscillatory activity differences are linked to resilience—and better behavioral performance in mice and humans

The current data show a better behavioral performance in neurocognitive domains of putative importance in resilience processes [9], namely, perceptual discrimination (pattern separation) in mice (Fig. 6) and cognitive control (aversive system inhibition) in humans (Fig. 2) in relation to phenotypic resilience and its neurophysiological correlates. By that, these data provide evidence that those 2 neurocognitive domains indeed are connected to resilience. However, the design of the human experiment does not allow to differentiate between the possibilities that resilience leads to better cognitive control or vice versa. Given the strong support for reappraisal as a resilience mechanism [9], a previous cognitive control training study led to improved reappraisal performance with better emotional control [47], indirectly suggesting that cognitive control may be able to enhance resilience. In our mouse model though, the comparison to the nonstressed control group (Figs. 5 and 6) cautiously indicates that resilience or at least the process of acquiring resilience is accompanied by an improvement of perceptual discrimination performance. This gain of behavioral functioning thereby constitutes a neurobiological example of posttraumatic growth [29] and/or stress inoculation [30]. Previous research also supports the idea that one of the resilience mechanisms might be a better discrimination ability [34–38].

### Macro- and microcircuit visual cortex network states of resilience as metastable, self-stabilizing, and noncontinuous entities on the neural systems level

Resilient outcomes in our translational model are related to noncontinuous network states as indicated in their distinct micro-neural (single-neuron activity) and macro-neural (β-/γ-band oscillatory activity) network activity. The temporal delay in the mouse model (see Microcircuit plasticity in the visual cortex may potentially be induced by a short-term neuronal mechanism in resilient mice), in addition, implies that those network states are also metastable and self-stabilizing. We have recently argued that neural networks attempt to achieve a state that is temporarily stable (a notion we refer to as the selfish network) independent of the aim of preserving long-term functionality in the context of brain diseases [48]. The present data may constitute a first instance of neural network state transitions in the context of a healthy, resilient response of brain to psychosocial stress, i.e., metastable, self-stabilizing, and noncontinuous neural network states of resilience. We have suggested that such network state transitions follow attractor-like dynamics and their formal assessment will enable us to develop new network-based interventions to foster resilience [48]. Importantly, our idea of a distinct entity of metastable, self-stabilizing, and noncontinuous neural network states of resilience must be seen on the backdrop of the notion of a bound brain [49–51], i.e., that cortical areas become dynamically bound into functional networks by synchronization in a task- and state-dependent way. Indeed, our translational data exactly imply such a functional network bound in a task- and state-dependent way consisting of prefrontal–occipital neural network states of resilience.

### Conclusion

These results suggest that there is an instance of resilience at the neural systems level that involves active, dynamic processes rather than being merely passive responses to stress including functional network regions such as the visual cortex formerly unattended in resilience research. The results also constitute a first example that neural network states of resilience are metastable, self-stabilizing, and noncontinuous entities that could serve as a target for new neural network interventions (e.g., targeted stimulation such as EEG-triggered transcranial magnetic stimulation (TMS) or γ entrainment using sensory stimulation [40,52] for fostering resilience).

## Methods

### Human EEG—participants

A total of 121 healthy human subjects participated in this dual-center study after providing written informed consent (59 in Frankfurt and 62 in Mainz). All participants were screened for MRI exclusion criteria, mental health status (Mini-International Neuropsychiatric Interview) [53], and handedness (Edinburgh Handedness Inventory) [54]. Due to technical failures during task performance, 4 participants were excluded, leaving 117 subjects for behavioral analysis. Thirteen additional participants were excluded from electrophysiological analysis due to issues with MRI data, including incomplete datasets (3 due to panic attacks, 1 due to pain, 1 due to size constraints, and 8 who withdrew consent) and 1 due to external EEG noise. Therefore, all presented electrophysiological analyses include data from 103 participants (65 females; mean age ± SD, 25 ± 6 years) who completed the study.

### Human EEG—questionnaires

After study inclusion and screening for MRI exclusion criteria (see above), participants completed questionnaires on demographic information (gender, date, and place of birth, family origin, psychological diseases within the family, family tree, marital status), health and lifestyle (physical and mental diseases, height and weight, blood pressure, medication, usage of internet, working conditions, income, family relationships, MRI compatibility), and their drug consumption [Fagerström for nicotine consumption and the alcohol use disorders identification test (AUDIT) for alcohol consumption] (secutrail, www.secutrail.com). Further questionnaires were the Trier Inventory for the Assessment of Chronic Stress (TICS), the Short Form 36 Health Survey Questionnaire, the General Health Questionnaire (GHQ-28), the Life events checklist from LHC (adapted from [55]), the Cognitive Emotion Regulation Questionnaire (CERQ), the Positive and Negative Affect Schedule (PANAS), the State-Trait Anger Expression Inventory (STAXI), the State-Trait Anxiety Inventory (STAI), the Barratt Impulsiveness Scale (BIS-11 [56]), the Behavioral Activation and Behavioral Inhibition Scales (BIS/BAS), the Edinburgh Handedness Inventory [57], and an Intelligence test (L-P-S Leistungsprüfsystem UT-3 [58]).

### Human EEG—experimental setup

The EEG study was conducted in 2 sites (Frankfurt and Mainz). Fifty-nine participants at site 1 and 62 participants at site 2 took part in the experiment. The experiment was conducted over 3 d: days 1 to 2 with 2 EEG measurements and day 3 with one functional magnetic resonance imaging (fMRI). On day 1, participants were screened for exclusion criteria and completed the questionnaires. Over the first 2 d, participants performed 4 behavioral tasks (2 tasks for each experimental day): the emotional Stop-signal task, emotional Recent probes task, Cognitive emotion regulation task, and emotional Flanker task. The order of the task was randomized across subjects. During the completion of the task, an EEG and a digitalized electrode localization were recorded simultaneously. On day 3, a resting state fMRI as well as structure T1 and T2 MRI measurements were performed on each participant. Only the EEG data from the emotional Flanker task and structural MRI (see source model) are used in this study. The EEG data for the emotional Flanker task have been used in [11]. Further details on electrode localization and MRI imaging parameters can be found in [11].

### Human EEG—task design

To assess cognitive processing under emotional distraction, a modified version of the Eriksen Flanker task was used, implemented in Presentation (v18.1, Neurobehavioral Systems). Each trial began with a fixation cross (1,000 ms), followed by an emotional image (500 ms) from the International Affective Picture System (IAPS), and then a Flanker stimulus (1,000 ms) consisting of white arrows on a black screen. Emotional images were either neutral or negative in valence. The Flanker stimulus included either congruent or incongruent flankers relative to the central target arrow. The inter-trial interval was 400 ms. Participants indicated the direction of the central arrow using the left or right Ctrl key with the respective index finger. Errors or slow responses (>1,000 ms) triggered a feedback message for 300 ms. A total of 1,120 trials were presented across 5 blocks (224 trials each; ~11 min per block) during EEG recording. Between blocks, participants had a 5-min break. Emotional stimuli included 280 neutral images (mean valence = 5.15, arousal = 3.17) and 280 negative images (valence = 2.47, arousal = 6.41), each shown twice with different Flanker combinations. This created 4 experimental conditions: Neutral Congruent, Neutral Incongruent, Negative Congruent, and Negative Incongruent. Each condition included 280 trials, balanced for left/right responses. Participants completed a 20-trial training block with neutral images before the main task. Additional information on task design can be found in [11].

### Human EEG—operationalization and measurement of resilience in human subjects

To quantify resilience in human participants, we computed an SR proxy score following the residualization approach described by Kalisch et al. [8]. In this method, mental health problems, measured via GHQ scores, are regressed on stressor exposure to capture individual deviations from the normative group-level relationship. Although the original approach combined Life events (LE) and Daily hassles (DH) as stressor measures, similar regression relationships have been demonstrated when only partial data are available [8]. As DH data were not available in the present dataset, stressor exposure was operationalized as the total number of LEs reported in the past 3 months. The residuals of the GHQ-on-LE regression served as the SR proxy score: Positive residuals indicated worse-than-expected mental health given stressor exposure (i.e., higher SR and lower resilience), whereas negative residuals indicated better-than-expected mental health (i.e., lower SR and higher resilience). To confirm that the retrospectively reported LE in our SR proxy score does indeed influence current mental health (GHQ), we applied a correlation analysis showing their significant association of the previous LEs to GHQ (*r* = 0.21; *P* = 0.03). Additional confirmatory analysis using weighted LE can be found in Text S1. This SR proxy score variable serves as a key metric in our analysis, allowing us to explore how individual resilience relates to both behavioral performance and network activity in the Emotional Flanker task.

### Human EEG—source reconstruction

The source reconstruction analysis was performed as described in detail in [11]. Briefly, individual finite element head models (FEMs) were created using the FieldTrip-Simbio pipeline, based on 5-tissue segmentation (skin, skull, cerebrospinal fluid, white matter, and gray matter). A template grid in MNI space was warped to each individual head model to enable direct comparison across participants. Source activity was estimated using the Dynamic Imaging of Coherent Sources (DICS) beamformer, with cross-spectral density matrices computed for baseline and task time windows in multiple frequency bands (θ, β, γ, high γ). Within-subject statistics were calculated using a dual-state beamformer approach, followed by a 2 × 2 repeated-measures cluster permutation ANOVA to test for effects of emotion and cognitive interference. Multiple comparison correction was applied across frequency bands, time windows, and grid voxels using Bonferroni and cluster-based permutation methods. Furthermore, the coordinates of the active sources were identified using the local maxima of the activation, without any prior assumptions about their locations. This methodology aligns with the best practice recommendations for source reconstructions in MEG [59]. For full methodological details, see [11].

### Human EEG—GC

The conditional GC (cGC) findings used in this study come from [11]. In the following, we report the main steps of the cGC analysis and statistical analysis; further details can be found in [11]. cGC was computed with a nonparametric variant of cGC. We obtained the CSD matrix of the reconstructed source activity after Flanker onset, using a fast Fourier transform in combination with multitapers (5-Hz smoothing) in 2-time windows: an early time window (0 to 250 ms) and a late time window (200 to 450 ms) to assure stationarity of the data. Additionally, we used a block-wise approach [60] considering the first 2 principal components (PCs) of each source stimulus as a block, then estimating the cGC that a source X exerts over a source Y conditional on the remaining areas [61]. We applied the cGC to the 3 IFG subdivisions (IFG_Tri_, IFG_Orb_, and IFG_Op_) and the posterior sources that showed significant interaction effects: V2 and precuneus (high γ band). We focused on bidirectional connectivity within IFG (IFG_Tri_, IFG_Op_, and IFG_Orb_) and of each IFG subdivision and V2 and precuneus, respectively. Only long-range significant cGC from IFG subdivisions to visual cortex is reported in this study. For each source–target pair, we performed a dependent-samples permutation ANOVA (α 0.05) with cluster-based correction across frequencies (cluster α 0.05). Multiple correction was applied using Bonferroni correction to correct for multiple source testing.

### Human EEG—Bayesian linear regression Granger

We employed a Bayesian linear regression to determine which of the significant links from IFG to visual areas (V2 and precuneus) predict the SR proxy score. For the *j*th subject, we define the likelihood of SR proxy score given by *y*, denoted as:

$$\begin{array}{c} y_{j}∼N\left(\alpha +\beta_{1}*\mathrm{cG}C_{j}^{\mathrm{lin}k_{1}}+\ldots +\beta_{n}*\mathrm{cG}C_{j}^{\mathrm{lin}k_{n}},\;\sigma^{2}\right) \end{array}$$ (1)

where, for the *j*th subject, α is the intercept and encodes the grand average of SR proxy score when all predictors are zero, $\mathrm{cG}C_{j}^{\mathrm{lin}k_{1,..,n}}$ denotes the cGC for subject *j* on link *n* (e.g., from IFG_Tri_ to V2). β_i_ represents the regression coefficients quantifying the relationship between the cGC links of the IFG subdivisions to precuneus or V2 and the dependent variables (SR proxy scores), and $\sigma^{2}$ represents the residual variance. The model consisted of 7 links. We *z*-normalized all variables before modeling, and β coefficients are reported with respect to these standardized variables. All parameters were assumed to be drawn from normal distributions. We used weakly informative priors in both models: The intercept and regression coefficients were assigned normal priors with mean 0 and standard deviation 0.5, and the residual standard deviation was given a half-normal prior with scale parameter 1.

### Human EEG—minimum and maximum power definition

To examine the relevance of oscillatory power interactions with SR proxy scores, we extracted, for each subject, the minimum and maximum power interaction values within predefined frequency bands and regions of interest. Specifically, β-band (10 to 44 Hz) interactions were assessed in the IFG, while γ-band (44 to 150 Hz) interactions were evaluated in the precuneus and V2. As interaction strength is proportional to the absolute deviation from zero, values closest to zero indicate the weakest interactions, whereas values with the largest magnitude (positive or negative) reflect the strongest interactions. Time–frequency representations (TFRs) of source-reconstructed EEG power were computed using FieldTrip [62]. For the β band, we employed a Morlet wavelet convolution approach, convolving the data in the 2- to 44-Hz range in 1-Hz steps [notice that minimum and maximum were computed only over the β-band (10 to 44 Hz) range]. The wavelet width varied linearly from 3 to 8 cycles across frequencies. For the γ band, we applied a multitaper convolution method using discrete prolate spheroidal sequences (DPSS), with power estimated in 2-Hz steps from 44 to 150 Hz. The length of the sliding time window was adjusted to ensure approximately 7 cycles per frequency, with spectral smoothing set to 20% of the center frequency.

### Human EEG—Bayesian linear regression min/max power interaction

To investigate the relationship between oscillatory power interactions and SR proxy scores, we conducted multiple Bayesian linear regression analyses. Each model used the SR proxy score as the dependent variable and evaluated the predictive value of power interaction features extracted from source-reconstructed time–frequency data. Specifically, 4 models were compared: (a) a model including only the minimum β-band power interactions in the IFG pars triangularis (IFG_Tri_), (b) a model including only the maximum β-band power interactions in the IFG pars triangularis (IFG_Tri_), (c) a model including only the minimum γ-band power interactions in V2, (d) a model including only the maximum β-band power interactions in the IFG pars triangularis (IFG_Tri_), (e) a combined model including the minimum β power in IFG_Tri_ and the maximum γ power in V2, and (f) a full model incorporating all 4 predictors (min/max β in IFG_Tri_ and min/max γ in V2). Bayesian model estimation and comparison were used to evaluate the evidence for each model in explaining inter-individual variability in SR proxy scores, allowing for quantification of model uncertainty and regularization of parameter estimates.

For the *j*th subject, we define the likelihood of SR proxy score given by *y*, denoted as:

Model 1: $y_{j}∼N\left(\alpha +\beta_{1}*\text{IF}G_{\mathrm{Tr}i_{j}}^{\min},\;\sigma^{2}\right)$

Model 2: $y_{j}∼N\left(\alpha +\beta_{1}*\text{IF}G_{\mathrm{Tr}i_{j}}^{\max},\;\sigma^{2}\right)$

Model 3: $y_{j}∼N\left(\alpha +\beta_{1}*V2_{j}^{\min},\;\sigma^{2}\right)$

Model 4: $y_{j}∼N\left(\alpha +\beta_{1}*V2_{j}^{\max},\;\sigma^{2}\right)$

Model 5: $\begin{array}{c} y_{j}∼N\left(\alpha +\beta_{1}*\text{IF}G_{\mathrm{Tr}i_{j}}^{\min}+\beta_{2}*V2_{j}^{\max},\;\sigma^{2}\right) \end{array}$,

Model 6: $\begin{array}{c} \begin{array}{c} \begin{array}{c} y_{j}∼N\left(\alpha +\beta_{1}*\text{IF}G_{\mathrm{Tr}i_{j}}^{\min}+\beta_{2}*FG_{\mathrm{Tr}i_{j}}^{\max}+\beta_{3}*V2_{j}^{\min}+\beta_{4}*V2_{j}^{\max},\;\sigma^{2}\right) \end{array} \end{array} \end{array}$

Each model describes the SR proxy score y for the *j*th subject as a function of oscillatory power interaction features. In these equations, αis the intercept, representing the expected SR proxy score when all predictors are zero—effectively encoding the grand average SR score across the population. The terms $x_{j}^{\min/\max}$ denote the power interaction predictors (e.g., minimum or maximum β power in IFG_Tri,_ or γ power in V2) for subject *j*. These predictors reflect the strength of frequency-specific interactions derived from source-reconstructed EEG signals. The coefficients $\beta_{n}$ are regression weights capturing the influence of each power interaction predictor on the SR proxy score. In the regression models, each coefficient $\beta_{n}$ quantifies the relationship between a specific power interaction feature and the SR proxy score. Since γ power interactions (e.g., in V2) are typically positive, a positive $\beta_{n}$ indicates that stronger γ interactions (i.e., larger positive values) are associated with higher SR proxy scores. Conversely, for β power interactions (e.g., in IFG_Tri_), which tend to be negative, a negative $\beta_{n}$ implies that more negative values—reflecting stronger β interactions—are associated with higher SR proxy scores. Thus, while the sign of the interaction values differs by frequency band, the interpretation of $\beta_{n}$ remains consistent: It reflects whether stronger interactions in that specific direction (positive or negative) are associated with increased or decreased SR. Finally, σ^2^ is the residual variance. All models were estimated with weakly informative priors on regression coefficients β∼N(0,1) and a Half-Cauchy prior on σ, unless otherwise specified.

### Human EEG—Bayesian regression setup and model comparison

We estimated the model regression coefficients using Bayesian inference with Markov chain Monte Carlo (MCMC) sampling, using the python package pymc3 [63] with NUTS (NO-U-Turn Sampling), using multiple independent Markov chains. We implemented 4 chains with 3,000 burn-in (tuning) steps using NUTS. Then, each chain performed 10,000 steps. Those steps were used to approximate the posterior distribution. To check the validity of the sampling, we verified that the R-hat statistic was below 1.05. To evaluate different models with different numbers of parameters, we implemented cross-validation, which has been advocated for Bayesian model comparison, e.g., in [64,65]. In particular, we adopted the leave-one-out cross-validation (LOO-CV) implemented in PyMC3. Lower LOO-CV scores imply better models. We report the full modeling and model comparison results in Table S1 and only include the results of the winning models in the main text.

### Human EEG—γ-burst analysis

To identify γ-burst events, we adopted the following pipeline. At first, we parcellated the visual cortex using the visual topography probabilistic map (VPTM) atlas. This parcellation consisted of 25 parcels per hemisphere. We constrained our source reconstruction to only parcels related to left and right V1 and V2. Spatial filters were concatenated across vertices comprising those parcels, and we obtained a set of time courses of the event-related field at each parcel. For each parcel, we selected the first spatial components explaining most of the variance in the signal. We adopted this method rather than averaging to avoid signal cancellation due to sign ambiguity of the reconstructed time course. To extract γ-burst time points, we adopted a method from [66], which showed direct good agreement between visual cortical spiking and presence of γ bursts on local field potential (LFP) data. In brief, γ bursts were identified using a generative model for oscillatory bursts. Source time-courses were bandpass-filtered in the 40- to 80-Hz range using a zero-phase finite impulse response (FIR) filter with a center frequency of 60 Hz and a filter order of 11. The signals were then modeled as a combination of spontaneous background activity and transient oscillatory bursts. A dictionary of 30 representative γ waveforms was learned from the data using sparse coding and correntropy-based dictionary learning. Since we performed this analysis on baseline data (i.e., pooled conditions), we used the first 100 trials for training. To detect burst events in the test data (remaining ~1,000 trials), each bandpass-filtered LFP trace was convolved with the learned dictionary atoms. Candidate bursts were then identified by applying an adaptive threshold to the resulting projection, based on the instantaneous power of the matched oscillatory components. IEIs between γ bursts were computed by measuring the time difference between the peaks of successive identified γ events. For consistency with the analysis performed on the animal data, we conducted a 2-sided Kolmogorov–Smirnov test to compare the distribution of IEIs between the resilient group (individuals with negative SR proxy scores) and the nonresilient group (positive SR proxy scores) over the pooled visual cortical areas.

### Animal—CSD and SI test

Mice were subjected to a CSD paradigm according to established protocols [12,13]. Mice were introduced into a home cage of an older, larger, and retired male CD1 breeder. During a physical exposure phase of 2 min, the CD1 mouse was allowed to attack the BL6 experimental mouse. For the consecutive sensory exposure phase, a mesh wall was introduced in the middle of the cage between the 2 mice, allowing sensory but not physical contact for 24 h. The procedure was reiterated for 10 d, and experimental mice were encountering different CD1 aggressors daily. On the last day of the CSD, all mice were housed individually in new cages and left to rest until the SI test took place 1 week later. The CD1 aggressors were trained for 3 d before beginning CSD to standardize attack’s latency. The group of nonstressed mice was handled throughout 10 d and placed for 1.5 min in an empty cage before they were returned to their individual cages separated in half by identical mesh walls. The SI test was performed 7 d later [12,13]. A mesh enclosure was presented at the center of an arena, and the interaction zone was defined as 1 cm. Mice were introduced twice into the arena for 2.5 min, first with an empty mesh enclosure, followed by re-introduction with a novel CD1 mouse under the enclosure. The SI score was calculated by forming the quotient of the dwell time of the BL6 mouse within the interaction zone, while a CD1 animal is in the mesh versus an empty mesh. The threshold value was chosen in accordance with Golden et al. [13], and animals with an SI value less than 100 are classified as nonresilient, while animals with an SI value greater than 100 are classified as resilient.

### Animal preparation

Mice were placed on a stereotactic frame (David Kopf Instruments, CA, USA) and anesthetized with isoflurane/oxygen (2% vol/vol) by inhalation while placed on a heat plate (ATC 2000, World Precision Instruments, FL, USA) to maintain body temperature at 37 °C. The skull was exposed and thoroughly cleaned from any remaining tissue. A craniotomy of 2 mm diameter was conducted at the coordinates of the primary visual cortex V1 (posterior −3 mm and lateral −2.5 mm to bregma) using a dental drill (Ultimate XL-F, NSK, Trier, Germany, and VS1/4HP/005, Meisinger, Neuss, Germany) under a dissecting microscope (Leica M80 stereo microscope, Leica, Wetzlar, Germany). For expression of the genetically encoded calcium indicator GCaMP6f via viral gene delivery, a total volume of approximately 1.0 μl of AAV1.CamKII.GCaMP6f.WPRE.SV40 solution was manually pressure injected with a 30° angle in 3 depths (150, 200, and 250 μm) into V1. The craniotomy was sealed with a transparent coverslip (3 mm diameter, 0.1 mm thickness) and glued to the skull using super glue (Vetbond, 3M, MN, USA). A ring-shaped head holder with an outer diameter of 14 mm and an inner diameter of 7 mm was implanted onto the skull using UV-glue (Polytec UV-Glue 2195, Polytec PT GmbH, Karlsbad, Germany) with the notch facing the rear of the animal. After the procedure, mice were allowed to recover for 3 weeks prior to habituation. A detailed description of surgical methods is available in [18].

### Animal habituation

Mice were habituated over 5 consecutive days to avoid stress exposure during imaging. On day 1, mice were handled for at least 15 min to get familiar with the experimenter. On days 2 and 3, mice were constrained after at least 15 min of handling by manually holding the head holder. On day 4, mice were constrained upon handling using the retaining device that is mounted below the microscope during imaging sessions. Mice were undergoing a mock experiment on day 5 by constraining them with the head holder and mounting them onto the spheric treadmill (JetBall-TFT, PhenoSys, Berlin, Germany) under the microscope. A 15-min recording session was simulated by activating the microscope but keeping all shutters closed to avoid photobleaching.

### Animal—in vivo awake 2-photon imaging

Mice were head fixated and mounted on a spheric treadmill, surrounded by a 270° screen system for presenting visual stimuli. In vivo recordings were conducted using a custom-built 2-photon microscope equipped with a resonance scanner (TrimScope II, LaVision Biotec, Bielefeld, Germany), and indicator excitation was achieved by a femtosecond pulsed Ti:sapphire laser (Chameleon II, Coherent Systems, CA, USA). A 40× water immersion objective [0.8 numerical aperture (NA); NIRAPO, Nikon, Tokyo, Japan] was used for imaging, resolving a field of view (FOV) of 277 × 277 μm represented in a matrix of intensity values with 512 × 512 pixel. Image acquisition was controlled by ImSpector Pro software (LaVision Biotec, Bielefeld, Germany) at a frame rate of 30.8 Hz. Relevant information from all subsystems were collected by a multichannel data acquisition interface (CED Power3, Cambridge Electronics, UK) and exported subsequently to each imaging session using the software package Spike2 (Cambridge Electronics, UK). We initially imaged the spontaneous activity of a neuronal microcircuit for 15 min in layer II/III of V1, followed by 12.5 min of visually evoked activity by employing a visual stimulation paradigm. The respective imaging depth of each animal can be found in Table S2. The paradigm consisted of initially 5 s of gray screen, followed by the presentation of 8 randomized static and drifting grating directions lasting 5 s each. The paradigm was repeated 10 times, every time with a novel randomization.

### Animal—imaging data analysis

All datasets were motion corrected to address *x*–*y* movement artefacts using the moco [67] plugin in FIJI ImageJ [68]. As reference image, an average intensity projection was calculated using the z-project function of ImageJ. We employed a custom-written semi-automated MATLAB (The MathWorks, Natick, MA, USA) script for segmentation of all visible neuronal somata within the FOV. The algorithm created an average intensity projection of all single images of the time series, and we marked each neuron with a polygon-shaped outline and defined them as regions of interest (ROIs). We then averaged all intensity values of all pixels within each ROI for every image of the series, resulting in an intensity trace per ROI. A 10-s-long period of quiescence free of calcium transients or signal fluctuations was defined as baseline F0 for each ROI separately. The relative change of fluorescence Δ*F*/*F* was calculated for each sample point *F* with [69]. In the next step, we exported the intensity traces to a custom-written IGOR Pro (Wavemetrics Inc., OR, USA) procedure to detect putatively AP-related calcium transients. First, intensity traces were smoothed by a Gaussian kernel 20 to 30 times, high-pass filtered with a pass band at 0.12/Fs, where Fs is the sampling frequency, and inspected for the presence of calcium transients. Intensity traces that did not exhibit transients or falsely identified structures were excluded from further analysis in this step. Second, a threshold-based algorithm automatically detected signal peaks exceeding 2.5 to 3 SD above the mean. Furthermore, the first and second derivatives were calculated, which had to be 0 and negative, respectively, to meet the criteria of an AP-related calcium transient. The typical decay of the calcium deflection was modeled by fitting an exponential curve using the CurveFit tool of IGOR Pro between the peak and the tail. If necessary, peak locations were corrected manually. Lastly, the onset of a given calcium transient was defined as the first data point prior to the identified peak that dropped below 0.5 SD, the offset when the fitted exponential curve reached 0.5 SD of the baseline. Intensity traces were binarized by representing periods of quiescence as 0 and the sample points of a transient from onset to offset as 1. The circular variance (CV) was calculated as the variance of the cell’s response to all orientations:

$$\mathrm{CV}=1-\left|\frac{\sum_{k}r_{k}e^{i2\theta_{k}}}{\sum_{k}r_{k}}\right|$$ (2)

where 𝑟_𝑘_ is the cell’s response to a given orientation and 𝜃_𝑘_ is the angle of the grating in radians [70]. To test for synchronicity, activity intervals for each neuron were refined by defining the onset and offset of each identified peak as the first frame to the left and right of the peak frame, respectively, that had a Δ*F*/*F* intensity ¡ 0.5 times peak intensity. Activity intervals were defined as all data points between onset and offset. Peaks occurring during an identified activity interval were not considered in a subsequent estimation of onset/offset but were instead considered part of the existing activity interval. The computation of Δ*F*/*F* traces in this step was adapted from the Allen Brain SDK 2.14 as a windowed median filter detrending, employing a long filter of 5,401 frames and a short filter of 101 frames (Allen Institute for Brain Science, 2022, Software Development Kit [2.14], available from https://allensdk.readthedocs.io/en/latest/). The binarized activity matrix of all ROIs was used to create pairwise correlations between each pair of ROIs to visualize the connectivity between neurons within timeframes in which at least one ROI showed activity. In the resulting connectivity map, neurons were depicted as nodes based on their *x* and *y* positions and were assigned a color value that corresponded to their activity frequency. Edges between nodes were represented as lines whose linewidth was defined by the achieved correlation value. Microscopy of cleared samples was performed in horizontal orientation on the light sheet microscope UltraMicroscope II (Miltenyi Biotec) with a 2×/0.5 NA objective (MV PLAPO 2XC, Olympus) with corrected dipping cap attached and the zoom factor of ×0.63, using the operating software Imspector Pro 7.7.0. Light sheet width was set to 100%, sheet NA—0.163 (3.9 μm thickness), merging algorithm—fixed blend, step size—3 μm, dynamic horizontal focus was used with 8 steps and fixed blending mode. Laser module beam combiner was used with separate laser channels: excitation, 640 nm; emission, 680 nm; 10% power; 100-ms exposure time. Acquired images were processed using arivis Vision4D (Carl Zeiss Microscopy Software Center Rostock GmbH, Germany). iDISCO+ tissue clearing was performed according to Pastore et al. [71] with modifications.

## Ethical Approval

### Human EEG—ethics information

The study and all experimental protocols were approved by the local ethics committees of the Medical Board of Rhineland-Palatinate, Mainz, Germany, and Johann-Wolfgang-Goethe-University, Frankfurt, Germany [ethical approval: 837.074.16(10393)], and all participants were financially compensated for study participation.

### Animal—ethic

All experiments were carried out along institutional animal welfare guidelines and were approved by the Landesuntersuchungsamt Koblenz, State of Rhineland-Palatinate, Germany. For experiments, male C57/BL6 mice were used. Mice were group housed until the beginning of the CSD paradigm. Afterward, animals were single-housed. During the whole experiment, mice had access to food and water ad libitum.

## Data Availability

Animal and anonymized human data supporting the findings of this study are available from the corresponding authors upon reasonable request.
