# Supplementary material for: A Translational Neural Network Mechanism of Resilience: Top-Down Control and Plasticity of the Visual Cortex Relates to Resilient Outcome and Performance
Source: Research (Wash D C). 2026 Apr 1;9:1215. doi: 10.34133/research.1215 (PMC13040084; doi:10.34133/research.1215)
Supplement: Supplementary 1 — Text S1 Figs. S1 to S3 Tables S1 and S2 [file research.1215.f1.docx]

# Supplementary material

**S1 Text: Confirmatory Analysis Using Weighted Life Event Scores**

As an additional confirmatory analysis, we examined the relationship between weighted life events (LEs)—which account for both the number and the perceived impact of past stressors—and current mental health (GHQ) scores. This analysis revealed a stronger and statistically significant correlation (*r* = 0.25, *p* = 0.006), further supporting the relevance of retrospectively reported LEs in explaining individual differences in current psychological distress.


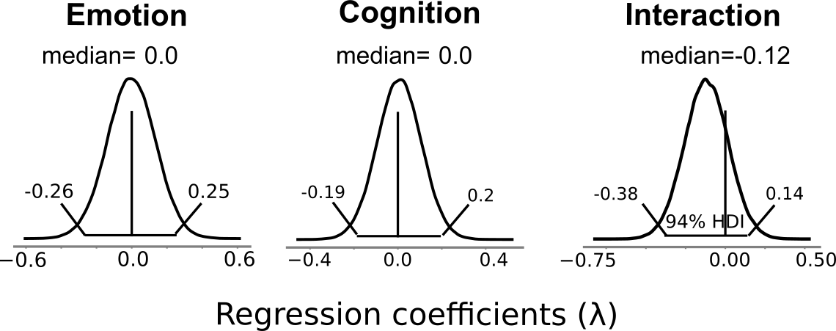


**S1 Fig SR score relates to SR score relates to behavioral measures.** Posterior distributions of the regression coefficients (λ) and their 94% highest density intervals (HDI), for the Bayesian linear regression of SR proxy scores against accuracy (ACC) differences for neutral versus negative emotional stimuli (ΔACC_emotion_) (left), congruent versus incongruent Flanker stimuli (ΔACC_cognition_) (middle), and the interaction effect in accuracy (ΔACC_interaction_ = (ΔACC_Incong_. _Neg._−ΔACC_Incong. Neut._) − (ΔACC_Cong. Neg._−ΔACC_Cong. Neut._)) (right).


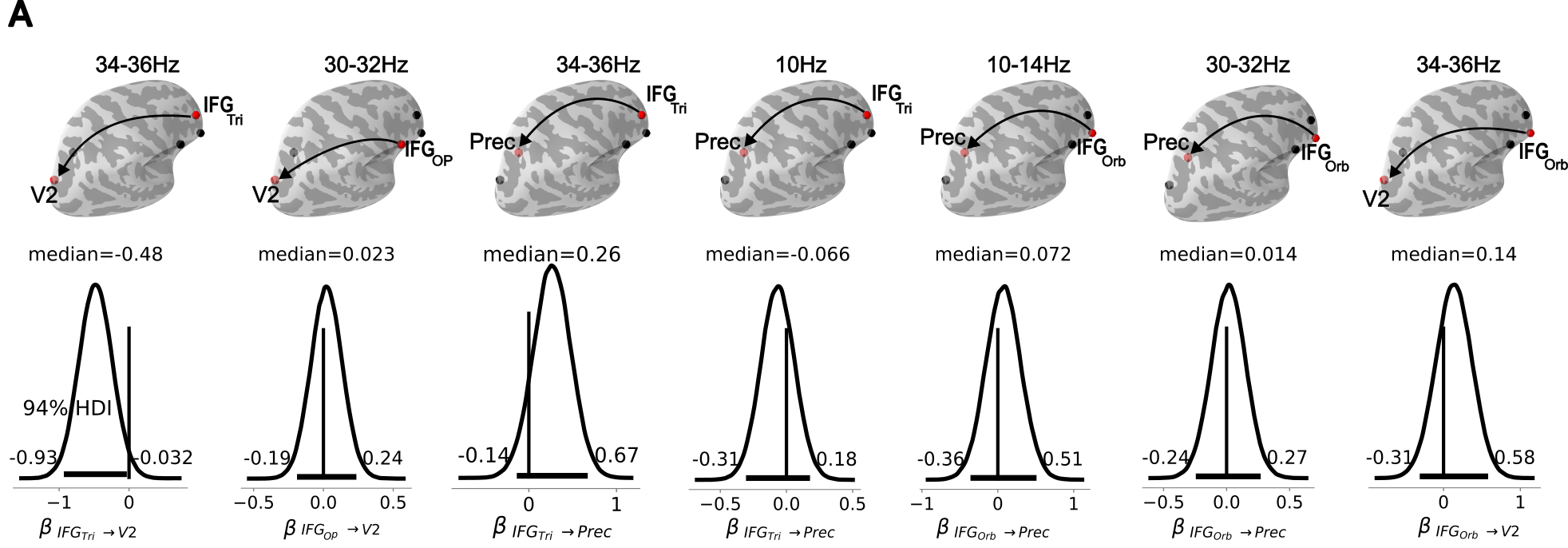


**S2 Fig SR proxy scores impact long range information flow and statistical interaction effects in spectral power at the source level for all seven GC links.** (A) Posterior distributions and 94%-HDI for Bayesian linear regression of SR proxy scores against various long-range information flows in multiple frequency bands (α, β, γ) assessed by Granger Causality (GC) from: IFG_Tri_ to V2, IFGOp to V2, IFG_Tri_ to Precuneus (34-36Hz), IFG_Tri_ to Precuneus (10Hz), IFG_Orb_ to Precuneus (10-14HZ), IFG_Orb_ to, Precuneus (30-32HZ) and IFG_Orb_ to V2.

Only the **IFG**_Tri_ **→ V2** connection showed a reliable effect, with the 94% highest density interval excluding zero, indicating a significant top–down influence.

**
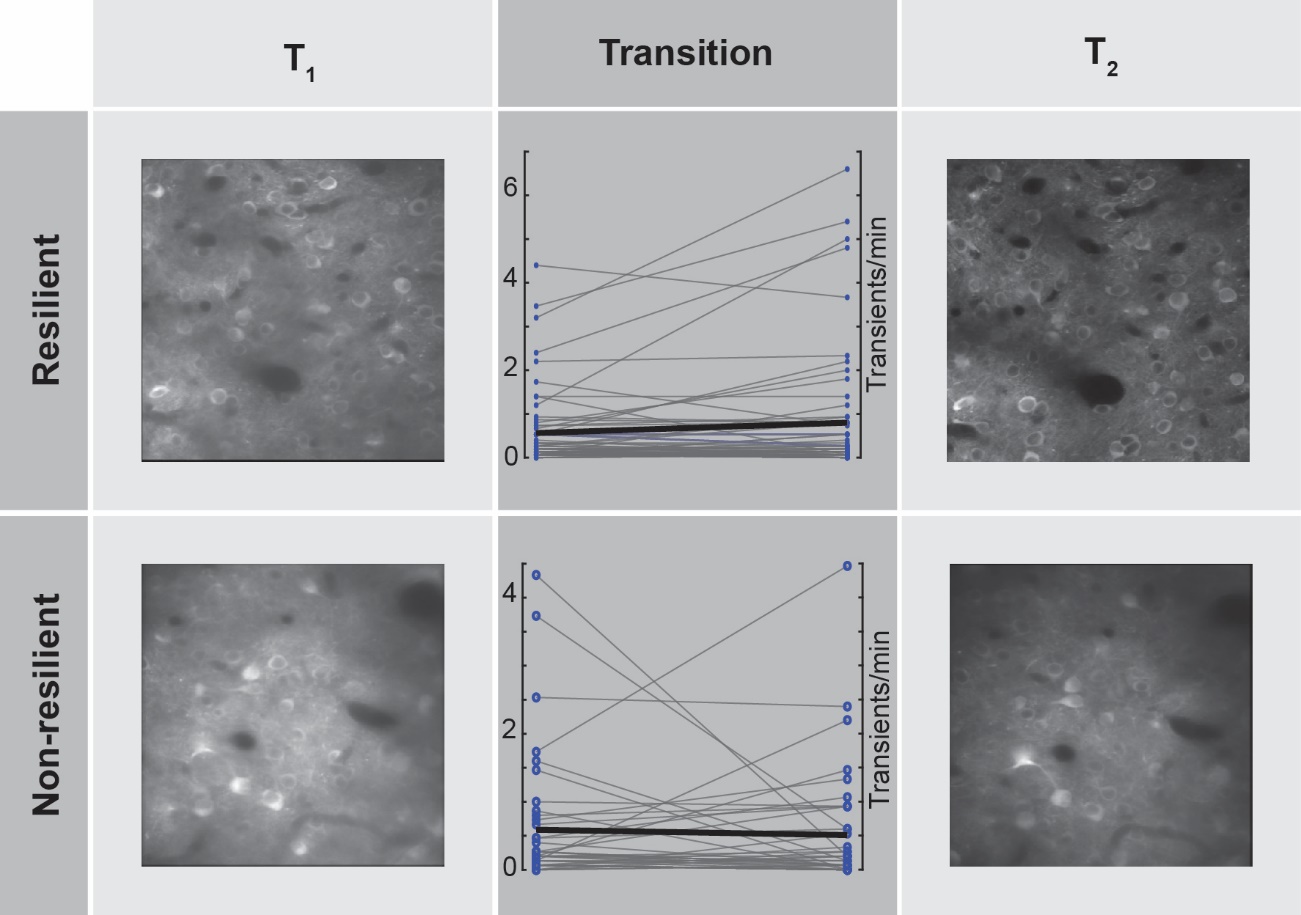
**

**S3 Fig Assessment of the long-term stability of the local network activity.** A two-photon micrograph of a resilient and a non-resilient animal, each measured at a one-week interval (T1, T2). The transition of the activity levels for all neurons that could be identified at both time points indicate shifts in firing frequencies of a subfraction of neurons. The cumulative distributions of all pooled neurons of the respective animal show no significant difference between the two imaging timepoints

**Supplementary table 1**

| MODEL NAME | ELPD LOO | P LOO | ELPD DIFF | SE | WEIGHT |
| --- | --- | --- | --- | --- | --- |
| **Model 5** | **-144.318276** | **3.548634** | **0.0** | **6.640720** | **0.8270** |
| Model 1 | -145.266765 | 2.850264 | 0.9484 | 6.755870 | 0.0 |
| Model 6 | -145.609408 | 5.142828 | 1.291131 | 6.632299 | 0.0 |
| Model 4 | -146.420400 | 2.521780 | 2.102123 | 6.805197 | 0.17 |
| Model 3 | -147.426991 | 2.357991 | 3.108714 | 6.950197 | 0.0 |
| Model 2 | -147.659097 | 2.832034 | 3.340820 | 7.054917 | 0.0 |

Leave-one-out cross-validation (LOO) results for Bayesian linear regression models predicting stressor reactivity (SR) proxy scores based on oscillatory power interaction features. Each model included different combinations of beta- and gamma-band power interactions extracted from source-reconstructed time–frequency data in V2 and the inferior frontal gyrus pars triangularis (IFG_Tri_). See subsection 5.12 for additional information. Reported are the expected log predictive density (ELPD LOO), effective number of parameters (P LOO), ELPD difference from the top model, standard error (SE), and stacking weight. **Model 5,** which included minimum beta power in IFG_Tri_ and maximum gamma power in V2, outperformed all other models in predictive performance and received the highest stacking weight (0.83), supporting its selection as the best candidate model.

**Supplementary table 2**

| Animal | Imaging depth [µm] |
| --- | --- |
| R1 | 205 |
| R2 | 193 |
| R3 | 294 |
| R4 | 278 |
| R5 | 170 |
| S1 | 207 |
| S2 | 193 |
| S3 | 161 |
| S4 | 158 |
| S5 | 271 |
| S6 | 280 |
| C1 | 172 |
| C2 | 230 |
| C3 | 153 |

Imaging depth relative to the cortex surface in micrometers. Recordings represent neurons in layer II/III in mouse visual cortex.
